# Discovery of signatures of fatal neonatal illness in vital signs using highly comparative time-series analysis

**DOI:** 10.1038/s41746-021-00551-z

**Published:** 2022-01-17

**Authors:** Justin C. Niestroy, J. Randall Moorman, Maxwell A. Levinson, Sadnan Al Manir, Timothy W. Clark, Karen D. Fairchild, Douglas E. Lake

**Affiliations:** 1 Department of Public Health Sciences, University of Virginia, Charlottesville, VA 22947 USA; 2 Center for Advanced Medical Analytics, University of Virginia, Charlottesville, VA 22947 USA; 3 Department of Medicine, University of Virginia, Charlottesville, VA 22947 USA; 4 School of Data Science, University of Virginia, Charlottesville, VA 22947 USA; 5 Department of Pediatrics, University of Virginia, Charlottesville, VA 22947 USA; 6 Department of Statistics, University of Virginia, Charlottesville, VA 22947 USA

**Keywords:** Neonatology, Applied mathematics

## Abstract

To seek new signatures of illness in heart rate and oxygen saturation vital signs from Neonatal Intensive Care Unit (NICU) patients, we implemented highly comparative time-series analysis to discover features of all-cause mortality in the next 7 days. We collected 0.5 Hz heart rate and oxygen saturation vital signs of infants in the University of Virginia NICU from 2009 to 2019. We applied 4998 algorithmic operations from 11 mathematical families to random daily 10 min segments from 5957 NICU infants, 205 of whom died. We clustered the results and selected a representative from each, and examined multivariable logistic regression models. 3555 operations were usable; 20 cluster medoids held more than 81% of the information, and a multivariable model had AUC 0.83. New algorithms outperformed others: moving threshold, successive increases, surprise, and random walk. We computed provenance of the computations and constructed a software library with links to the data. We conclude that highly comparative time-series analysis revealed new vital sign measures to identify NICU patients at the highest risk of death in the next week.

## Introduction

Continuously monitored vital signs of patients in intensive care units hold untapped information about risk for adverse events and outcomes^1^. For example, the display of a score based on analysis of abnormal heart rate characteristics was shown by our group to reduce sepsis-associated mortality by 40% in preterm infants in the Neonatal Intensive Care Unit (NICU)^2–5^. That approach was tailored to detect specific phenomena that we observed in the heart rate data, reduced variability and transient decelerations, in the days prior to sepsis diagnosis^2–5^, and we used algorithms optimized for the task, including sample asymmetry^6^ and sample entropy^7–9^.

Here, we asked a more general question—what if we did not know all the characteristics we wish the algorithms to detect? That is, if we used a very large number of algorithms designed for general use in time-series, would we discover some that were more effective than our tailored design? This approach has been described by Fulcher et al., who called it *highly comparative time-series analysis*^10–12^. The fundamental idea is to extract features from many time series, using many algorithms, most operating with many sets of parameter values. We then apply this ensemble to a dataset to determine which algorithms perform best for predicting a specific outcome. Clustering of algorithms can, eventually, simplify this approach for clinical applications^13,14^.

As an example of our approach, the familiar sample entropy algorithm^7,8^ requires two parameters in order to operate, an embedding dimension *m* and a tolerance window *r*. A highly comparative time-series analysis entails many operations of the sample entropy algorithm that vary *m* and *r*. The result of each operation is treated as a potential predictor. Since the results are expected to be highly correlated, we can represent the family of sample entropy results as a cluster, and choose an operation of sample entropy with a single optimal combination of *m* and *r* for use in multivariable statistical models.

Furthermore, rather than simply clustering methods we know to be in the same family (“sample entropy” etc.), but with differing parameters, we can expand clustering to include many families of methods, and their parameters, defining the clusters using an outcome similarity measure. The cluster that contains sample entropy might then also contain related measures detected by clustering, all of which can be represented by a single outcome measure or feature. In all, this is an efficient way to screen many time-series algorithms, to discover features that are predictive of an outcome, without domain knowledge of prior known specific characteristics such as reduced heart rate variability, that might be related to that outcome.

To test these ideas, we selected death in the next 7 days for infants in the NICU as the outcome of interest. This is a topic of clinical interest and usefulness—identification of infants at high risk, especially where risk appears to be rising quickly, can alert clinicians to the possibility of imminent clinical deterioration from illnesses such as sepsis or respiratory failure. The heart rate characteristics score noted above, which is targeted toward a specific time-series phenotype, has modest performance in this area^15^. In this work, the question is whether an examination—and potentially a combination—of many time series feature extraction algorithms may improve on this targeted approach.

## Results

### Patient population and causes of death

From January 2009 to December 2019, 6837 infants were admitted to the UVa NICU, with median gestational age (GA) 35 weeks. Of these, 5957 infants had heart rate and oxygen saturation data available for analysis, and 205 died and had heart rate and oxygen saturation data available within 7 days of death. Table 1 gives the demographics of the patient population and Table 2 gives the causes of death. For 152 of the 205 infants that died, support was redirected due to critical illness and grim prognosis. Of these, 148 died within minutes to hours after removal from mechanical ventilation. The other 4 infants died 2–4 days after the ventilator was discontinued, during which time comfort measures were provided. The remaining 53 infants died while on mechanical ventilation. In all cases, full support was provided while the infants were on mechanical ventilation.

**Table 1:** Demographics of the patient population.

Cohort	ALL	Survived	Died
	5957 (100%)	5752 (96.6%)	205 (3.4%)
Gestational age mean weeks (SD)	34.6 (4.5)	32.4 (5.9)	34.7 (4.4)
Birth weight mean kg (SD)	2.460 (1.001)	2.475 (0.992)	2.030 (1.156)
Extremely preterm (n% <28 weeks)	576 (10%)	515 (9%)	61 (30%)
Sex (%female)	43%	43%	42%
Race (%Black)	19%	19%	19%
Ethnicity (%Hispanic)	7%	7%	7%
Mechanical ventilation mean days (SD)	4.9 (19.8)	4.6 (19.4)	14.1 (17.3)
Seizures^a^	4%	4%	14%
Congenital cardiac malformation^b^	9%	9%	18%
Genetic syndrome or multiple anomalies	4%	4%	15%

*HIE* hypoxic ischemic encephalopathy.

^a^Confirmed by electroencephalogram.

^b^Excluding septal defects and patent ductus arteriosus.

**Table 2:** Causes of death.

Primary cause of death categories	ALL	Extreme prematurity^a^	Brain disorders	Congenital cardiac disease	Multiple congenital anomalies^b^	Lung disease, pulmonary hypertension	Sepsis, Necrotizing Enterocolitis	Other^c^
*n* (%)	*n* = 205 (100%)	*n* = 26 (13%)	*n* = 37 (18%)	*n* = 23 (11%)	*n* = 33 (16%)	*n* = 38 (18%)	*n* = 28 (14%)	*n* = 20 (10%)
Gestational age (mean weeks)	32.4	24.7	36.2	36.3	34.7	31.1	29.2	33.9
Birth weight (mean kg)	2.03	0.735	2.952	2.895	2.044	1.705	1.447	2.405
Sex (%female)	42%	35%	41%	32%	43%	29%	46%	55%
Race (%Black)	19%	27%	16%	13%	18%	21%	7%	35%
Ethnicity (%Hispanic)	4%	4%	3%	4%	12%	0%	4%	0%
Age at death (mean)	24.3 days	2.1 days	10.9 days	10.6 days	22.2 days	54.6 days	32.0 days	29.0 days
Event Days	871	64	149	87	140	182	153	96
HR-SpO_2_ Top 5+Demographic	0.853	0.913	0.915	0.894	0.900	0.875	0.744	0.774
HR-SpO_2_ Top 5	0.828	0.911	0.823	0.899	0.837	0.860	0.759	0.779
Demographic	0.714	0.709	0.897	0.713	0.823	0.721	0.512	0.607

HR-SpO_2_ Top 5: Statistical model using the top 5 measures of heart rate and O_2_ saturation.

Demographic: Statistical model using birth weight, gestational age, sex and 5 min Apgar score.

HR-SpO_2_ Top 5+Demographic: Statistical model using all the above.

Values are AUCs.

^a^<29 weeks gestation with respiratory failure and/or severe intraventricular hemorrhage.

^b^Including genetic syndromes.

^c^Including hydrops, metabolic disorders, renal or liver failure, and unknown.

### AUC for death prediction for each of the 3555 operations

**Fig. 1:**
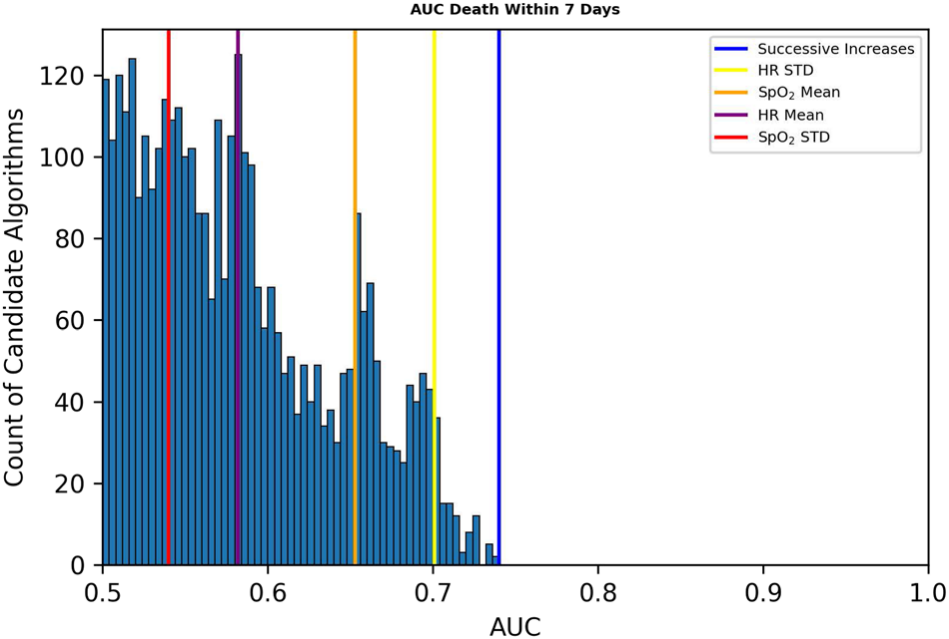
AUCs of 3555 operations for predicting death in the next 7 days. Colored vertical bars from left to right indicate the AUCs of the standard deviation of oxygen saturation, mean heart rate, mean oxygen saturation, standard deviation of heart rate, and a novel measure, successive increases of heart rate.

In total, there were 871 daily 10 min samples within a week of death for 205 infants, for a sample incidence rate of 0.67%. Figure 1 shows the number of algorithms as a function of their univariate predictive performance for death in the next week, measured as AUC. The top performing algorithm, a symbolic logic count of successive increases in heart rate that is discussed further below, had an AUC of 0.799, substantially higher than that of the traditional algorithms like standard deviation of heart rate (0.749) and mean oxygen saturation (0.639).

### Algorithmic results clustered, allowing data reduction

**Fig. 2:**
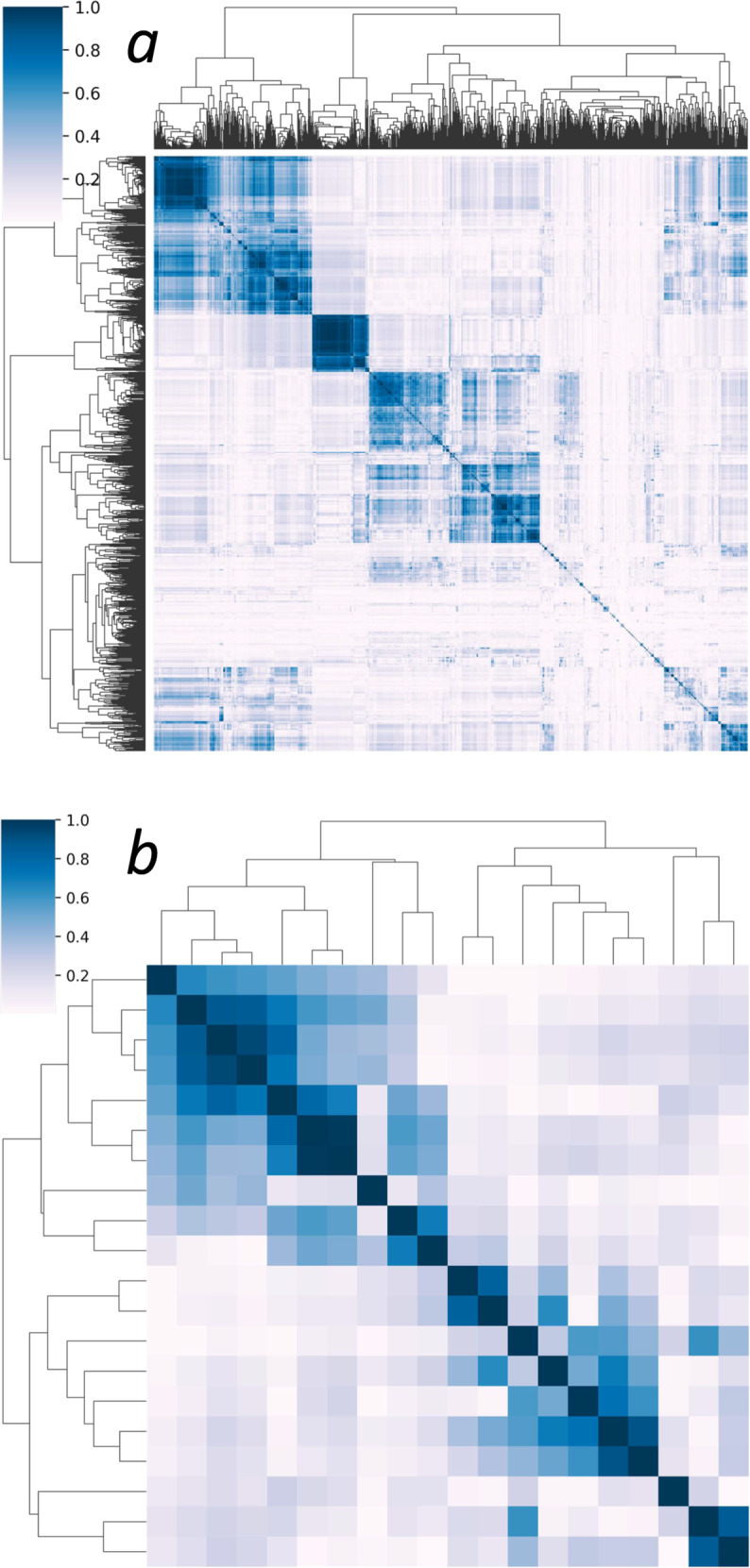
Heat maps of the absolute values of the correlation coefficients between results of operations. **a** Correlations between all 3555 candidate algorithmic operations. **b** Correlations between 20 cluster medoids. The reduced feature set explains 81% of the variance in the full set.

**Fig. 3:**
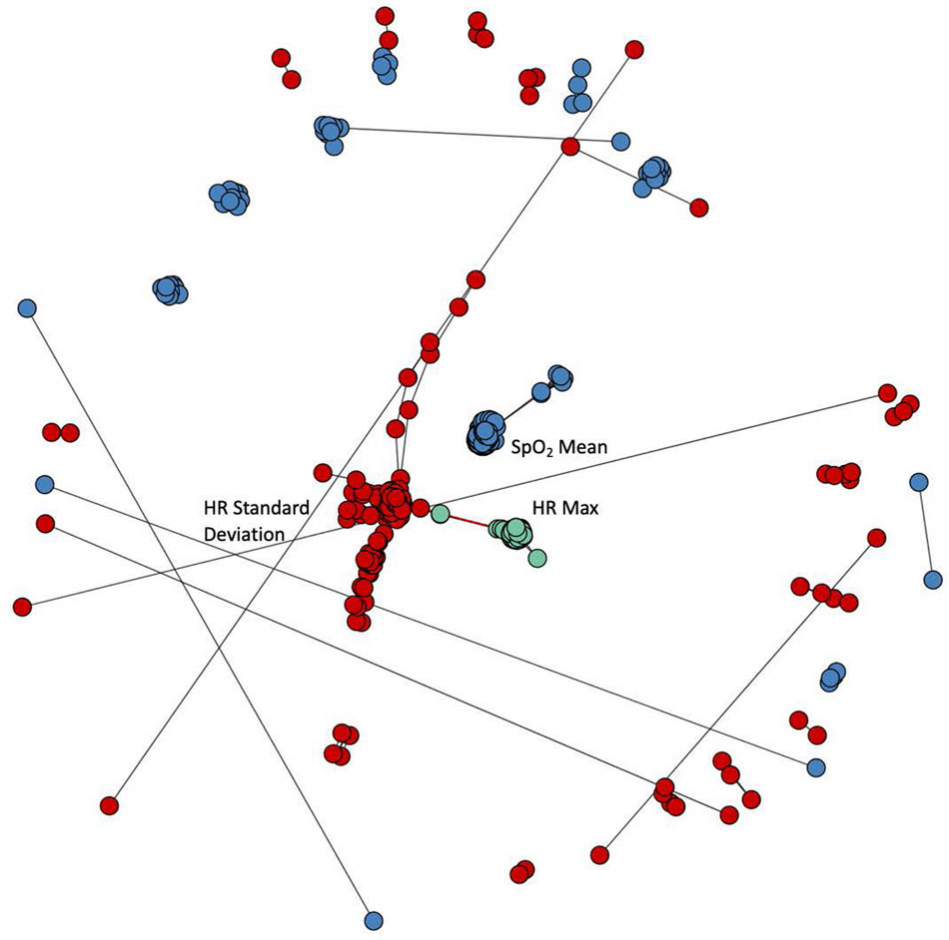
Three representative clusters of operations and their relations to each other. Each dot represents an individual operation. The colors—green, blue, and red—represent the three clusters; several individual operations are labeled. The green group represents measures of the maximum of the heart rate, the blue group reports on the mean oxygen saturation, and the red group reports on the standard deviation of the heart rate. The black lines indicate pairings between operations in the same cluster, whereas the very short red line indicates the small number of pairings between operations from different clusters. heart rate heart rate; STD standard deviation.

We sought correlations among the results. Figure 2 shows two heat maps based on the absolute value of the correlation coefficients for 3555 algorithmic operations on the left and 20 identified by cluster medoids on the right. These results justified an analysis of clusters of results, which we undertook by measuring mutual information among all the operational results. A representative cluster is shown in Fig. 3. We sought a number of clusters that was large enough to explain most of the predictive performance of a multivariable statistical model for death but in keeping with the practice of having a reasonable number of predictors for 200 events^16,17^. We found that 20 clusters satisfied these conditions, and selected the top-performing operation in each as the representative. The findings were robust in repeated experiments with different random sampling of one record per patient per day as well as daily averaged data.

We examined the clusters for interpretability, and found that clusters of algorithmic operations reported on identifiable and interpretable time-series characteristics. For example, one cluster held the results of operations that report on the mean, another cluster held operations that report on the minimum, and so on. As expected, very near neighbors represented the results of operations that are closely related. To reduce the dimensionality of the data, we represented each of the 20 clusters by a single, interpretable operation.

### Multivariable statistical models using the new measures of vital signs to predict death

To understand the association of mathematical operations to the outcome of death, we used multivariable logistic regression. We selected features using backwards selection and were conservative, restricting the model size to a limit of five features. Each patient was represented on each day by 20 candidate features—10 for each heart rate and each oxygen saturation time-series—calculated on a 10 min record chosen at random once per day. In order to reduce the effects of possible outliers, results were winsorized by clipping low and high values of the results at 0.1% and 99.9% respectively.

To better understand how these models performed leading up to the day of death, AUCs for each model were calculated daily from 7 days to 1 day prior to death, as shown in Table 3. For example, the AUC at 7 days was calculated excluding all data from the 6 days prior to death and only using the model output between 6 and 7 days. This excludes many deaths in first week of life and others that might be have been expected.

**Table 3:** Model performances as a function of days until death.

Model name	Candidate features	Model size	≤7 days	≤ 1 day	3 days	7 days
HR-SpO_2_ - demographics	21	6	0.853	0.903	0.819	0.774
HR-SpO_2_	20	5	0.828	0.892	0.794	0.747
HR-SpO_2_	20	3	0.821	0.887	0.781	0.742
HR-SpO_2_: cluster centers	20	5	0.819	0.873	0.790	0.763
HR	10	5	0.809	0.864	0.770	0.750
HR: successive increases	1	1	0.799	0.858	0.755	0.746
HR-SpO_2_: means and SDs	4	4	0.774	0.816	0.733	0.731
SpO_2_	10	5	0.765	0.818	0.752	0.694
Demographics	4	4	0.714	0.710	0.683	0.697

AUC for models using a single random daily 10 min segment of heart rate (HR), oxygen saturation (SpO_2_), or both are shown at various intervals before death.

*HR* heart rate, *SpO*_*2*_ oxygen saturation, *SDs* standard deviations.

As expected, model performance was highest one day prior to death. The combined heart rate and oxygen saturation model with five features was one of best performers, with AUC of 0.892 the day before death and 0.747 a week prior to death. A model limited to five heart rate features had an AUC of 0.809 compared to the best heart rate univariate model, successive increases, which had an AUC of 0.799. The oxygen saturation only model limited to 5 features had an AUC of 0.765. The combined heart rate and oxygen saturation model limited to five features had an AUC of 0.828—this was the best-performing five-feature model. The AUC decreased slightly to 0.821 when limited to only three features. As a comparison, a combined heart rate and oxygen saturation model that was selected using AIC had 13 variables had a slightly improved AUC of 0.834.

A clinically relevant measure is accuracy of the model at a threshold equal to the event rate of 0.67%. For this case, the sensitivity is the same as the positive predictive value (PPV). For rare events it is informative to look at the ratio of this value to the event rate, or lift. The heart rate model had a sensitivity/PPV of 11.1% at this threshold for a lift of 16.5.

We tested how much discriminating capability was retained when we used the medoids of the 20 clusters versus the top performers in each. A combined heart rate and oxygen saturation model with five features from the 20 medoids had an AUC of 0.821, comparable to that obtained using the top performers. We conclude that each cluster can be reproducibly represented by a single operation.

Model performance can be improved by including baseline demographic information. A model consisting of birth weight, GA, sex and 5 min Apgar score had an AUC of 0.714, and 5 min Apgar score was the most predictive feature. Adding this demographic model to the combined heart rate and oxygen saturation model with five features increased the AUC from 0.828 to 0.853.

### New measures of vital signs associated with NICU death

We found new measures of heart rate and oxygen saturation signals associated with NICU deaths. In the heart rate time-series, the top performing measures were fewer occurrences of *successive increases* and larger *surprise*. In the oxygen saturation time-series, the most predictive measure was a *moving threshold* calculation^18^ that showed fewer extreme events was informative in both the heart rate and the oxygen saturation time-series. An algorithm fitting a random walk model to the oxygen saturation time-series detected declines in the oxygen saturation.

**Fig. 4:**
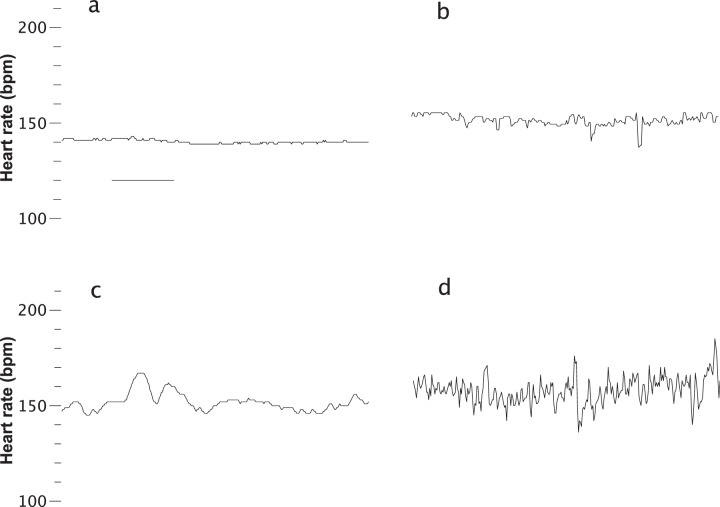
Lack of successive increases in heart rate predict increased risk of death. Four 10 min heart rate records that correspond to increasing death risk with a decreasing number of *successive increases* in heart rate. The record in **a** had no successive increases and reflected a 20.6-fold increased risk of death. **b**–**d** Show records with 15, 30, and 60 successive increases in heart rate and reflected fold-increased risks of death 3.5, 1.1, and 0.6, respectively.

The most informative new predictor for death risk was a small number of successive increases in the heart rate, and Fig. 4 shows four records with increasing numbers of successive increases. The value that would be observed in a set of 300 random numbers, 75 (300/0.5^2^), is approached in the lower right panel. Qualitatively, the finding is that low heart rate variability is associated with higher risk of death. However, a more direct measure of variability, the standard deviation of the heart rate, was less predictive (AUC 0.799 for successive increases compared with 0.749 for heart rate STD).

## Discussion

Much progress has been made in the use of continuous time-series data from the bedside continuous cardiorespiratory monitors in the Neonatal ICU^19^. We tested the idea that we might improve the current art through a systematic study of our very large set of time series using an exhaustive number of analytical measures. We draw from a prismatic work describing the method of highly comparative time-series analysis, applying many time series algorithms to many time series examples of all kinds^10^. We applied the principles of highly comparative time series analysis to our domain, continuous cardiorespiratory monitoring in NICU patients. This work extends the study of highly comparative time-series analysis with its focus on clinical datasets, clinical events that are important to clinicians, and domain-specific knowledge of the physiologic origins of the data and how clinicians use it at the bedside.

In this example of highly comparative time-series analysis of a large clinical dataset, we studied vital sign data from Neonatal ICU patients and discovered algorithms not previously reported in this domain that identified infants at higher risk of death. These algorithms report generally on the absence of heart rate variability and low oxygen saturation, features known to inform on poor health. Other major findings were that only 20 clusters of algorithms explained the great majority of the variance, in keeping with another study of highly comparative time-series analysis^14^, that downsampling of the data to single 10 min records daily did not affect the overall results, and that the newly revealed algorithms outperformed standard measures of vital sign variability.

We found that a small number of clusters explained the variance of the results. This is not entirely unexpected, because many of the operations entail the same algorithm repeated with different arguments and parameters. For example, the sample entropy algorithm requires the choice of an embedding dimension *m* and a tolerance window *r*^7–9^. In all, we performed 12 sample entropy operations with combinations of these variables. Thus, our clusters were to some extent explainable. For example, one held many operations that report on the center of the distributions, another held many reporting on the width of distributions, another held many entropy operations, and so on. These findings are important with regard to the interpretability of statistical models that use the results, avoiding the problem of black boxes^20^.

We found that downsampling of the data to single 10 min records daily did not affect the overall results for algorithmic operations clustering. A downside to the massive exploration of algorithmic operations is the computing time. To begin our investigation, we accordingly massively reduced the dataset to a single 10 min record daily, <1% of the total. To test the fidelity of the results, we repeated the procedure on 3 other single 10 min records, and on the daily average. The results were not significantly different in the nature of the clusters or their constituents, suggesting that a manageable subsample of the data can be used for exploratory purposes in the highly comparative time-series analysis, and the results verified afterward.

We found that newly revealed algorithms outperformed canonical measures of vital sign variability. Importantly, they were interpretable in the light of domain knowledge about neonatal clinical cardiovascular pathophysiology.

*Successive increases* in heart rate is the result of a symbolic dynamics analysis^21^, and represent small accelerations. Individual vital sign measurements are replaced by symbols that reflect whether they have increased, decreased, or stayed the same compared to the preceding measurement. Our finding was that the number of consecutive increases in every-2 s heart rate was reduced in the time series of infants at higher risk of death, as illustrated in Fig. 4. This finding is consonant with reduced heart rate variability, a known marker of abnormal clinical status. It is interesting to speculate why the absence of successive increases should improve upon ordinary measures of variability for prediction of death.

**Fig. 5:**
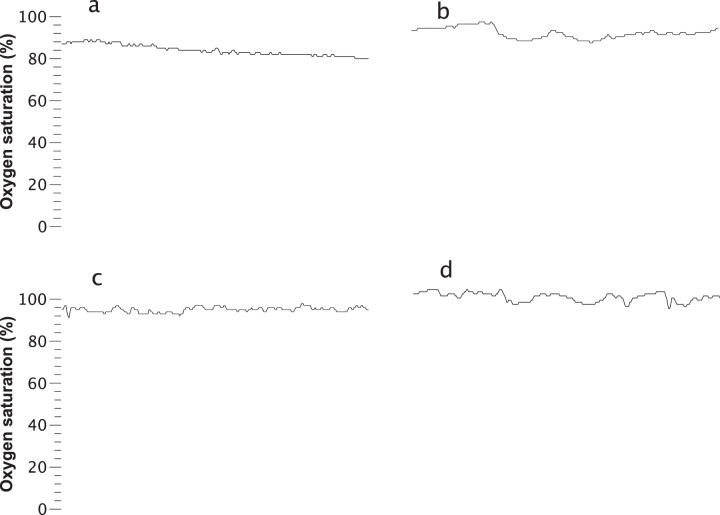
Lower moving threshold in oxygen saturation predicts increased risk of death. Four 10 min heart rate records that correspond to increasing death risk with a decreasing percentile value for oxygen saturation moving threshold. The record in (**a**) was the 0.5 percentile value and reflected tenfold increased risk of death. **b**–**d** Show records with moving threshold percentiles 10, 50, and 75, and fold-increased risks of death 2.3, 0.6, and 0.4, respectively.

*Moving threshold*^18^ was an approach developed in the field of extreme events in dynamical systems. An example is the human reaction to floods in rivers—when there are no floods, barriers are allowed to lapse. A flood, though, leads to new, higher barriers that could contain the recent event. The moving threshold model examines a time series for points that exceed an initial threshold, increases the threshold after a crossing event, and allows the threshold to decay over time. The parameters that are measured include the rate of events, the intervals between threshold crossings, and statistical measures of the distribution of the thresholds. Our finding was that the vital sign time series of infants at higher risk of death were characterized by lower moving threshold for heart rate (reflecting low heart rate variability) and lower moving threshold for oxygen saturation (illustrated in Fig. 5 as a gradual decline in SpO_2_).

*Surprise*^22^ calculates the distribution of points of a subsection of the time series. The surprise of the point following that subsection is measured by how likely the new point was given the calculated distribution, given as 1/*p*. The phenotype associated with mortality here, was a low value of surprise in the heart rate, consistent with reduced heart rate variability.

*Random walk model* measures the fit of the time-series data to a random walk^23^. The random walk starts at 0 and takes a step of size proportional to the distance from the previous point. The algorithm returns many statistics about the movement of the random walk and its relation to the original time series. The phenotype of high risk of death detected by this algorithm is a decline in the oxygen saturation.

We can relate the new finding to prior work. In 2001^24^, we showed that heart rate characteristics of low variability and transient decelerations added information to clinical findings quantified by the SNAP (Score for Acute Neonatal Physiology)^25^ and NTISS (Neonatal Therapeutic Intervention Scoring System)^26^ in the early detection of sepsis. A heart rate characteristics index predicted sepsis and all-cause mortality in preterm NICU patients^2,15,24,27^. We found that the AUC for the heart rate characteristics index developed at the University of Virginia and tested at Wake Forest University was 0.73. Subsequently we broadened the analyses to include conventional measures of heart rate and oxygen saturation in the first week after birth which we showed predict mortality among preterm NICU patients better than the validated and commonly accepted SNAPPE-II score (Score for Neonatal Acute Physiology-Perinatal Extension)^28–30^. and combined heart rate and SpO_2_^31^. In 2010^32^, Saria and coworkers showed that short- and long-term variability of heart rate and oxygen saturation in the first 3 h of life were useful in classifying premature infants at risk for high-morbidity courses. In the current work we showed that running an enormous number of operations on a single daily random 10 min window of heart rate and oxygen saturation data uncovered new measures that predict mortality better than our prior models.

How might these findings lead to future improvements in neonatal care? We point to the increasing use of Artificial Intelligence and Machine Learning using Big Data to provide predictive analytics monitoring for early detection of subacute potentially catastrophic illnesses. While the data sources remain the same—continuous cardiorespiratory monitoring, lab tests, and vital signs measurements—the analytical methods are growing in number. An unresolved question has been whether the identification of signatures of illness by domain experts can be replaced by exhaustive computer analysis of large datasets^33^. These new findings point clearly to a role for highly-comparative time series analysis to detect previously unthought-of ways to characterized the pathophysiological dynamics of neonatal illness. Future work will test these new candidate algorithms against existing ones of heart rate characteristics analysis^34^, cross-correlation of heart rate and oxygen saturation^35,36^, heart rate variability^37^, and others.

We acknowledge limitations. First, we did not analyze preterm infants separately from term infants in this work, though we know that heart rate and SpO_2_ time series characteristics depend on both GA and post-conceptual age. For example, the variabilities of heart rate and SpO_2_ rise with day of age^38,39^, and it is possible that highly-comparative time series analysis of preterm infants might return different results from term infants. As it stands, there were many more term infants than pre-term, but the latter represented more of the time series data. Second, external validation will be important because our findings of patterns in vital signs measurements prior to neonatal death might reflect care practices at our hospital. We note, though, the similarity of vital signs measurements at our hospital to those at two others^38^, a finding that is reassuring with regard to the general nature of these results. Third, we found that 1443 of the 4998 algorithms consistently returned null results, and we note that other datasets from other sources might fare differently. This was expected as many of the algorithms were from different domains and may not work on all signals. Finally, we used only logistic regression to test the association of the algorithmic operations with clinical outcome, and other machine learning and deep learning methods might have had different results. We note, though, recent works that point to a similarity of results of logistic regression compared to other methods including recurrent neural networks^40,41^.

We conclude that highly comparative time-series analysis of clinical data reduced thousands of algorithms to an interpretable set of 20 that represent the character of neonatal vital signs dynamics.

This framework will be useful for future work analyzing bedside monitor data for signatures associated with various imminent or future adverse events and outcomes. The terabytes of vital sign data at even just single NICU such as ours, together with electronic medical record data on clinical and laboratory variables, hold valuable insights into actionable outcomes. Developing platforms and systems for sharing data with other investigators so that algorithms can be tested in large and diverse populations is another worthy goal. Harnessing these data could lead to preemptive strategies that improve patient outcomes.

## Methods

### Study design

We collected all bedside monitor data from all patients in the Neonatal ICU (NICU) at the UVA Hospital since 2009 using the BedMaster Ex^TM^ system (Excel Medical, Jupiter FL). Heart rate derived from the bedside monitor electrocardiogram signal is sampled at 0.5 Hz. oxygen saturation is measured using Masimo SET^®^ pulse oximetry technology (Masimo Corporation, Irvine CA) with a sampling rate of 0.5 Hz and averaging time of 8 s. For this analysis, we included all infants admitted from 2009 to 2019 who had heart rate and oxygen saturation data available for analysis. Clinical data were abstracted from a NICU database (NeoData, Isoprime Corporation, Lisle, IL). The University of Virginia Institutional Review Board for Health Science Research approved a waiver of informed consent under DHHS regulations because the chart review was minimal risk, did not affect the subjects’ welfare, and was not otherwise practicable.

### Software and computing environment

We prepared a library of software in Python3 consisting of our implementations of 111 published algorithms^10^ described for use in medical and non-medical domains. Table 4 shows the families of algorithms employed, together with a description and examples of each.

**Table 4:** Families of algorithms implemented in highly comparative time series analysis.

Family	Description	Example(s)
Distribution	Moments and other descriptive statistics	Mean, median, standard deviation
Correlation	Similarity of data points as a function of the time between them	Linear and nonlinear autocorrelation
Stationarity	Statistical properties do not change over time	Standard deviation of moments measured on different window lengths
Symbolic transforms	Convert ranges to letters and analyze their sequence	Frequency of successive increases
Entropy	Order and regularity	Sample entropy
Trend analysis	Fitting lines through data	Slope and intercept
Heart Rate Variability	Canonical analyses	Power spectral density ratios
Time Series Modelling	Fits time series model to data	Surprise
Wavelet	Properties of the time series wavelet spectrum	Wavelet decomposition of time series
Nonlinear Analysis	Nonlinear analysis methods	False nearest neighbors, Information dimension
Other	Extreme values	Moving threshold model

We ran these routines and some additional special-purpose MATLAB algorithms in Docker containers designed to run in a horizontally scalable secure cluster environment under the OpenStack cloud operating system, using the FAIRSCAPE data lake environment^42^. We issued persistent identifiers for all software, datasets and analysis results using Archival Resource Keys (ARKs)^43^, associated with computational provenance metadata^44^ for reproducibility and reusability.

### Terminology: Features, algorithms, and operations

A *feature* of a vital signs time series is a pattern or phenotype that can be represented mathematically. For example, we speak of the features of heart rate time series before neonatal sepsis as abnormal heart rate characteristics of reduced variability and transient decelerations. *Algorithms* are the mathematical tools we use to quantify the features. For example, the standard deviation of the times between heartbeats quantifies the finding of reduced heart rate variability in illness. *Operations* further specify the details of algorithms. For example, the standard deviations of the times between heartbeats over the past 5 s or 5 min or 5 h all quantify heart rate variability, but they will return different values and, possibly, be of different utility clinically. The goal of highly-comparative time series analysis is to seek new features by widely exploring the spaces of algorithms and operations.

### Mathematical analysis of vital signs

**Fig. 6:**
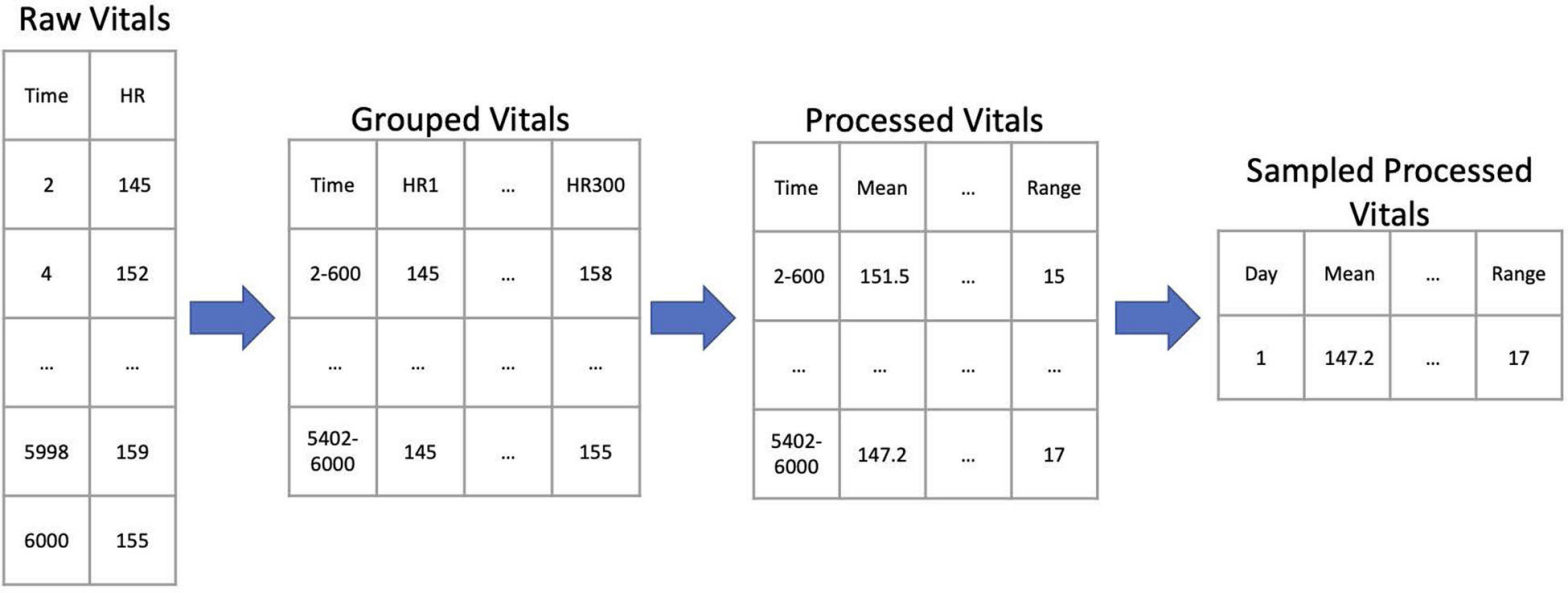
Data processing workflow for heart rate data. From left to right: Each row of the *Raw Vitals* table contains measured vital signs at 2 s intervals. These values are transposed in the *Group Vitals* table so that each row has a 600 s time range and up to 300 measured values. Algorithms operate on the *Group Vitals* table producing the *Processed Vitals* table of the same size—in the example, the first algorithm is the mean, and the last is the range. The *Averaged Processed Vitals* table holds the average of each result for a day; the *Sampled Processed Vitals* table holds the results for a randomly selected 600 s record.

The raw vital signs data were stored as vectors of time stamps 2 s apart with the corresponding measurement of heart rate or oxygen saturation. We grouped the vital signs data into more than 18 million 10 min non-overlapping windows, each with 300 measured values. In each group, we computed 81 time-series algorithms with varying parameters for a total of 2499 operations. The result was a matrix of results with more than 18 million rows and 2499 columns, as illustrated in the workflow diagram in Fig. 6.

We randomly sampled the *Processed Vitals* dataset taking one 10 min record per day per patient. This step resulted in 130,000 days of samples, each containing the result of 4998 operations from the heart rate and oxygenation data. We removed single-valued results, those with imaginary numbers, and samples with missing values, and were left with 3555 of the 4998 viable candidate algorithmic operations. To adjust for the wide range in scales, we used an outlier-robust sigmoid transform^10,45^ to convert operation ranges to the interval [0,1].

**Fig. 7:**
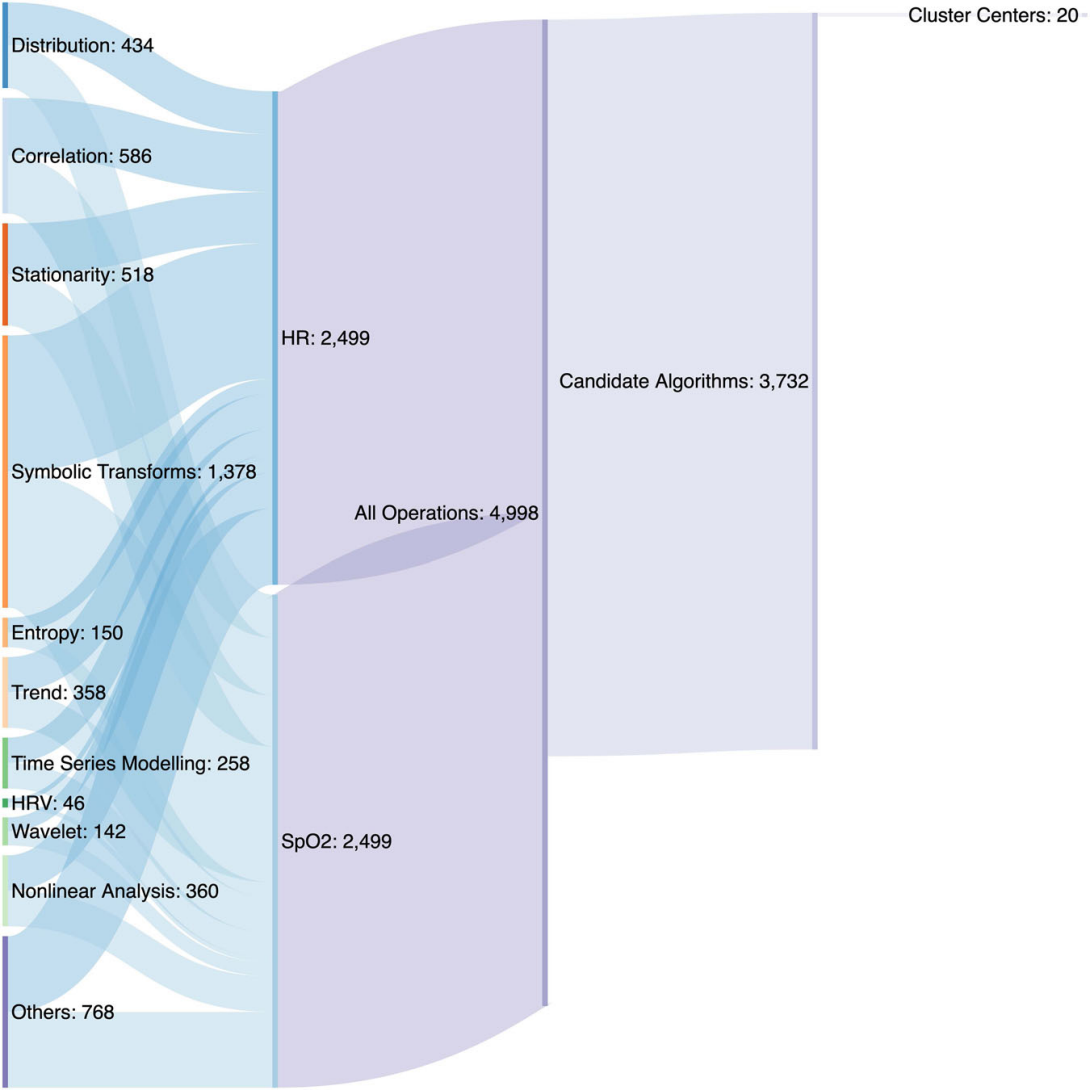
Sankey plot of the computational approach. Beginning with 3555 operations representing 11 families of algorithms, we ended with 20 clusters each represented by a single operation.

We clustered results to reduce dimensionality. We divided the 130,000 results of individual algorithms into ten equiprobable bins and calculated all possible distances using mutual information^46,47^. We organized these results into a distance matrix and determined clusters with k-medoids using the *pam* function of the R *cluster* package^48^. We represented each cluster by a single operation, as shown in Fig. 7.

### Statistical analysis and modeling

The binary outcome of death within the next 7 days was used to evaluate algorithm performance. Since there were 205 deaths, we restricted the number of clusters to 20, and selected the top performers in each as candidate features for model selection. This follows recommendations to use no fewer than 10 events for each predictor variable^16^. Several feature selection strategies were used including lasso, greedy stepwise selection, AIC, and all-subset logistic regression. For simplicity and to be extra conservative to prevent over-fitting, we decided to concentrate on models with no more than five features. A stepwise backwards procedure was used that started with a full logistic regression model and sequentially removed features with largest *p* value until there were five features. The performance of the model was calculated as the AUC using tenfold cross-validation.

### Reporting summary

Further information on research design is available in the Nature Research Reporting Summary linked to this article.

## Supplementary information

Reporting Summary

## Data Availability

Anonymized data that support the findings of this study, with the evidence graph for the clustering, are openly available in the University of Virginia’s LibraData archive at 10.18130/V3/VJXODP.

## Code availability

Python code used to process this data is archived in Zenodo at 10.5281/zenodo.4321332. This version and any future versions are also available in Github at https://github.com/fairscape/hctsa-py. Our code is licensed under terms of the MIT license (https://opensource.org/licenses/MIT), and is a reimplementation in Python of most of Ben Fulcher’s original MATLAB code, available here: https://github.com/benfulcher/hctsa. Software for clustering analysis and cross-implementation testing, together with the test data, may be found here: 10.5281/zenodo.4627625.
